# β-Cells Different Vulnerability to the Parkinsonian Neurotoxins Rotenone, 1-Methyl-4-phenylpyridinium (MPP^+^) and 6-Hydroxydopamine (6-OHDA)

**DOI:** 10.3390/ph14080767

**Published:** 2021-08-04

**Authors:** Marco Carli, Francesca Vaglini, Eleonora Risaliti, Gianluca Citi, Matilde Masini, Shivakumar Kolachalam, Roberto Maggio, Giovanni Umberto Corsini, Michela Novelli, Vincenzo De Tata, Marco Scarselli

**Affiliations:** 1 Department of Translational Research and New Technologies in Medicine and Surgery, University of Pisa, 56126 Pisa, Italy; francesca.vaglini@unipi.it (F.V.); e.risaliti@studenti.unipi.it (E.R.); gianciti@hotmail.it (G.C.); matilde.masini@unipi.it (M.M.); k.shivakumar@alumni.sssup.it (S.K.); gucorsini@tiscali.it (G.U.C.); michela.novelli@unipi.it (M.N.); vincenzo.detata@med.unipi.it (V.D.T.); 2 Department of Biotechnological and Applied Clinical Sciences, University of L’Aquila, 67100 L’Aquila, Italy; roberto.maggio@univaq.it; 3 C.I.M.E. (Centro Interdipartimentale di Microscopia Elettronica), University of Pisa, 56126 Pisa, Italy

**Keywords:** Parkinson’s disease, type 2 diabetes, neurotoxins, pesticides, rotenone, metformin, α-tocopherol

## Abstract

Neurotoxins such as rotenone, 1-methyl-4-phenylpyridinium (MPP^+^) and 6-hydroxydopamine (6-OHDA) are well known for their high toxicity on dopaminergic neurons and are associated with Parkinson’s disease (PD) in murine models and humans. In addition, PD patients often have glucose intolerance and may develop type 2 diabetes (T2D), whereas T2D patients have higher risk of PD compared to general population. Based on these premises, we evaluated the toxicity of these three toxins on pancreatic β-cell lines (INS-1 832/13 and MIN6) and we showed that rotenone is the most potent for reducing β-cells viability and altering mitochondrial structure and bioenergetics in the low nanomolar range, similar to that found in dopaminergic cell lines. MPP^+^ and 6-OHDA show similar effects but at higher concentration. Importantly, rotenone-induced toxicity was counteracted by α-tocopherol and partially by metformin, which are endowed with strong antioxidative and cytoprotective properties. These data show similarities between dopaminergic neurons and β-cells in terms of vulnerability to toxins and pharmacological agents capable to protect both cell types.

## 1. Introduction

Parkinson’s disease (PD) is a progressive neurodegenerative disorder of the central nervous system (CNS) that affects predominately the dopaminergic neurons of the *substantia nigra* and whose etiology can be either genetic or caused by environmental factors such as toxins, pesticides or other unknown agents [1]. Type 2 Diabetes (T2D) is a chronic metabolic disorder characterized by hyperglycemia, insulin resistance and decreased β-cell function, where in the long-term, β-cells eventually decline in terms of vitality and viability, diminishing in terms of number and density [2]. T2D usually begins with peripheral insulin resistance, leading to a supraphysiological increase of pancreatic insulin release that in the long-term has a detrimental effect of β-cell physiology. However, nowadays, it is well accepted that both aspects are relevant for the onset and progression of T2D [3,4,5]. 

PD patients often have glucose intolerance and they may develop T2D, whereas T2D patients have higher risk of PD compared to general population, underlying possible common pathological mechanisms, which explain the high level of comorbidity between these two disorders [6]. Among these mechanisms, inflammation, oxidative stress, mitochondrial dysfunction, susceptibility to environmental toxins and viruses have been proposed for both [7].

In addition, epidemiological findings have shown a higher prevalence of T2D and PD in rural communities exposed to pesticides and herbicides [8] and a list of potentially harmful environmental agents for pancreatic islets is growing continuously [9,10,11].

Intriguingly, pancreatic β-cells and dopaminergic neurons share several molecular elements making these two cell types more similar than expected. Human and rodent β-cell lines express dopamine receptors (D_1_–D_5_) [12,13,14] and enzymes for dopamine production (tyrosine hydroxylase and aromatic amino-acid decarboxylase) and degradation (MAO-A and B) [15]. With these premises, a physiological autocrine/paracrine function of dopamine for a fine regulation of β-cells insulin secretion has also been proposed [14]. Dopamine can be produced locally by its precursors originating from the diet, stored in insulin vesicles via vesicular monoamine transporter-2 (VMAT-2) and then secreted together with insulin [16,17]. β-cells also express transporters for dopamine (DAT) and its amino-acids precursors (LAT1 and 2) [15,18]. The presence of DAT and other transporters in β-cells could make them vulnerable to exogenous toxins similar to dopaminergic neurons. In addition, β cells are extremely sensitive to oxidative stress due to a high production of reactive oxygen species (ROS) and a low expression of endogenous antioxidants [19].

Among several toxins, rotenone, 1-methyl-4-phenyl-1,2,3,6-tetrahydropyridine (MPTP) and 6-hydroxy-dopamine (6-OHDA) have been extensively studied for their toxicity on dopaminergic neurons in vivo and in vitro; however, their effect on β-cells viability has not been properly considered [20]. These compounds, though structurally different, are grouped as the most common toxins used to induce PD in animal model experiments for altering the bioenergetics and viability of dopaminergic cells [21].

The correlation between exposure to rotenone and increased risk of PD in the general population is difficult to determine, but some meta-analysis have demonstrated an increased incidence of neurodegenerative diseases in rural communities exposed to rotenone [22,23]. MPTP was correlated to PD in the 1980s, when Parkinson-like phenotypes were found in young drug abusers who were unknowingly exposed to this toxin, a byproduct formed during illegal drug synthesis [24].

Rotenone is a widely used natural broad-spectrum insecticide and fish poison. Being highly lipophilic, it easily crosses the cell membranes and inhibits the activity of mitochondrial complex I. It determines the block of oxidative phosphorylation [25] and the flow of electrons to oxygen thereby increasing the production of reactive oxygen species (ROS) [26]. Other possible mechanisms of toxicity are associated to interference with PI3K/Akt/mTOR-related signaling [27] and microtubules dynamics [28].

MPTP is a contaminant found in illicit drugs and it is transformed into its active metabolite 1-methyl-4-phenylpyridinium (MPP^+^) by MAO-B. MPP^+^ accumulates in dopaminergic neurons through DAT and it is subsequently taken up by mitochondria where it directly inhibits mitochondrial complex I [29,30,31]. MPP^+^ has also been used as a broad-spectrum herbicide under the name of cyperquat that is structurally related to the well-known paraquat [32], which despite structurally similar is less neurotoxic than MPP^+^. 

6-OHDA, a neurotoxic synthetic organic compound, has been found in human brain and is taken up by catecholaminergic neurons via DAT and norepinephrine transporter (NET) [33]. The cellular toxicity is caused by inhibition of mitochondrial complex I and IV. In addition, it rapidly autoxidizes to para-quinone and H_2_O_2_ [21]. Though it has no use as a chemical compound for human activities, it is utilized as a prototypic pro-oxidant neurotoxin in PD models.

In this in vitro study, we aimed to test the toxicity of rotenone, MPP^+^ and 6-OHDA on pancreatic β-cells (INS-1 832/13 and MIN6) and described functional and structural changes associated to the exposure of these toxic compounds. 

We also tested several compounds known for their cytoprotective and antioxidant properties, such as the antidiabetic drug metformin and α-tocopherol, to evaluate whether they could counteract the toxic effects in β-cells, as previously reported for dopaminergic neurons [34,35].

## 2. Results

### 2.1. Effect of Rotenone, MPP^+^ and 6-OHDA on β-Cells Viability

**Figure 1:**
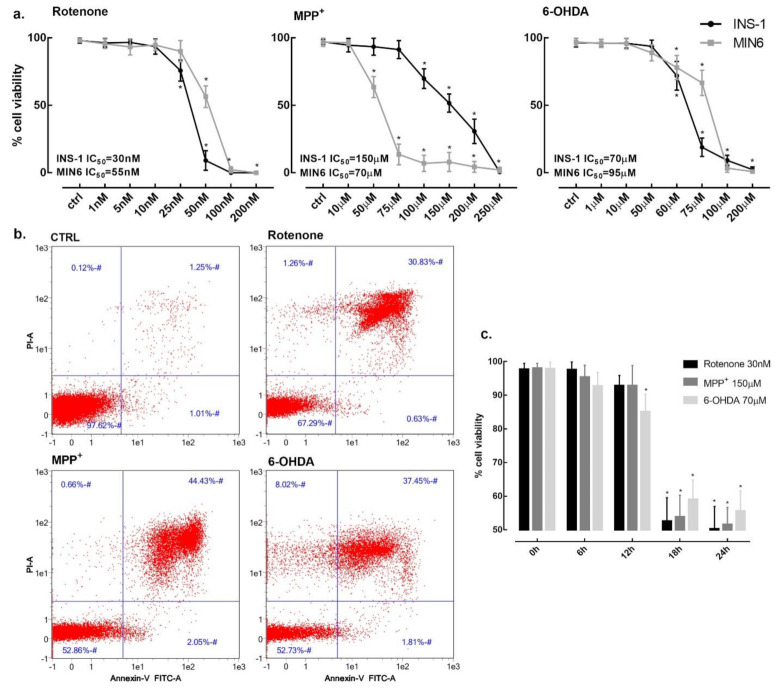
β-cells viability in the presence of rotenone, MPP^+^ and 6-OHDA. (**a**) INS-1 832/13 (black line) and MIN-6 (grey line) cells viability was assessed after 24 h exposure to increasing concentrations of rotenone, MPP^+^ and 6-OHDA. (**b**) Flow cytometric analysis of INS-1 cells viability. Cell death was determined by exposing cells for 24 h at the concentrations of 25 nM for rotenone, 150 μM for MPP^+^ and 70 μM for 6-OHDA. X and Y axis show Annexin V and propidium iodide (PI) staining markers, for apoptosis and necrosis, respectively. Each plot is divided into 4 regions. Lower-left region shows cells negative for both Annexin V and PI (living cells); lower-right region shows cells positive for Annexin V only (early apoptotic cells); upper-left region shows positive for PI only (necrotic cells); upper-right region shows positive for both (late apoptotic and necrotic cells). (**c**) Time course of INS-1 cells viability in the presence of 30 nM rotenone, 150 μM MPP^+^ and 70 μM 6-OHDA. Data are expressed as mean ± SEM of four independent experiments. * *p* < 0.05 compared to control, ANOVA, Dunnett’s *post hoc* test.

INS-1 832/13 (INS-1) rat and MIN-6 mouse insulinoma cells were treated with increasing concentrations of rotenone, MPP^+^ and 6-OHDA to evaluate their toxicity. Cell viability was carried out by using trypan blue exclusion assay after 24 h incubation and non-exposed cells were used as a control. Rotenone proved to be the most potent toxin with a IC_50_ of 30 nM and complete cell mortality was observed at concentration as low as 100 nM in INS-1. In MIN-6, rotenone IC_50_ was 55 nM. MPP^+^ showed a higher IC_50_ of 150 μM and 70 μM in INS-1 and MIN6, respectively, while the 6-OHDA IC_50_ was 70 μM for INS-1 and 95 μM for MIN6 (Figure 1a).

Given the similarity in terms of toxicity on both cell lines, we conducted all the subsequent experiments on INS-1. Cell viability was also determined by flow cytometry with Annexin V and propidium iodide (PI) staining markers to differentiate apoptotic, necrotic and healthy cells. After 24 h incubation, rotenone (25 nM) induced 30.3% cell mortality with only 0.5% of apoptotic cells, MPP^+^ (150 μM) induced 47.1% cell mortality with 2.0% of apoptotic cells and, finally, 6-OHDA (70 μM) induced 47.3% cell mortality with 1.8% of apoptotic cells (Figure 1b).

Cell death was not immediate and cell viability was not affected at 6 h and 12 h. Cell survival decreased between 12 h and 18 h for all three toxins at their respective IC_50_ concentrations (Figure 1c).

### 2.2. Effect of Rotenone, MPP^+^ and 6-OHDA on Glucose-Stimulated Insulin Secreting Function of INS-1 β-Cells

**Figure 2:**
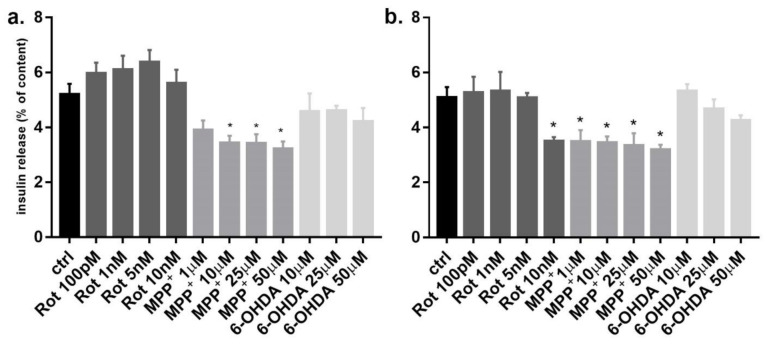
Effect of rotenone, MPP^+^ and 6-OHDA on INS-1 β-cells glucose stimulated insulin release. INS-1 cells were incubated for 6 h (**a**) and 24 h (**b**) at high glucose concentration (11 mM) in the presence of non toxic increasing concentration of rotenone, MPP^+^ and 6-OHDA. Insulin secretion was measured and expressed as percentage of the cellular insulin content. Data are expressed as mean ± SEM of three independent experiments. * *p* < 0.05 compared to control, ANOVA, Dunnett *post hoc* test.

To verify whether these three toxins could impair functioning of INS-1 before inducing cytotoxicity, we evaluated glucose stimulated insulin secretion (GSIS) function at low non-cytotoxic concentrations. After 6 h incubation, MPP^+^ at 10 μM was sufficient to induce a reduction of GSIS and this effect was confirmed at 24 h (Figure 2a). Rotenone clearly reduced GSIS after 24 h at the concentration of 10 nM, whereas 6-OHDA did not have any effect at 6 and 24 h up to 50 μM concentration (Figure 2b).

Since the concentrations of the three toxins were non-cytotoxic, the reduction of insulin secretion was not related to cell death. Moreover, the total cellular insulin content was not significantly different between treated and untreated cells (data not shown), indicating a normal insulin synthesis with impaired insulin secretion.

### 2.3. Effect of Rotenone, MPP^+^ and 6-OHDA on INS-1 β-Cells Mitochondrial Function

**Figure 3:**
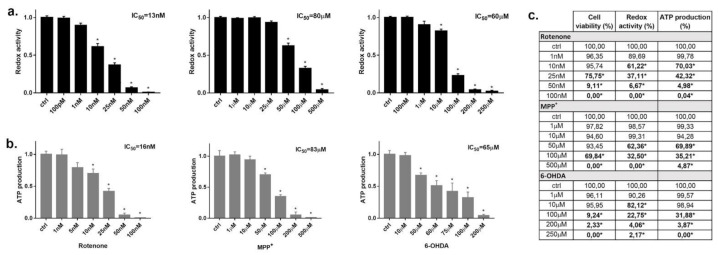
Effect of rotenone, MPP^+^ and 6-OHDA on INS-1 β-cells mitochondrial function. Changes of mitochondrial redox activity (**a**) and ATP production (**b**) in INS-1 cells treated for 24 h to increasing concentrations with the three toxins. (**a**) Redox activity was evaluated as difference in absorbance values for cells treated with the toxins compared to control after incubation with alamarBlue Reagent. (**b**) Cellular ATP content was evaluated as difference in luminescence values for cells treated with the toxins compared to control by using ATPlite assay. Data are expressed as mean ± SEM of four independent experiments and normalized over control signal. * *p* < 0.05 compared to control, ANOVA, Dunnett’s *post hoc* test. (**c**) % of reduction in the presence of the three toxins in the different assays (cell viability, redox activity and ATP production) are confronted in terms of cell death and mitochondrial inhibition. * bold *p* < 0.05 compared to control, ANOVA, Dunnett’s *post hoc* test.

Since these three neurotoxins are traditionally known to be mitochondrial respiratory chain inhibitors, we investigated whether they alter the mitochondrial function in INS-1 cells using alamarBlue redox indicator (redox activity). The alamarBlue assay is usually considered a marker of cell death; however, results from this test should be carefully considered for compounds directly inhibiting mitochondria activity. In fact, the block of mitochondrial activity could happen independently or before cell death [36]. Indeed, rotenone inhibited the transformation of the redox dye in a dose-dependent manner (Figure 3a) with an IC_50_ of 13 nM. On this matter, we could verify by comparing with trypan blue experiments that only a small proportion (~4%) of cells were dead at rotenone’s IC_50_ in the alamarBlue assay (Figure 3c). Similar evidence was found for MPP^+^, where the IC_50_ in the alamarBlue assay was 80 μM and, at the same concentration, cell viability was over 90% (Figure 3a,c). For 6-OHDA, the IC_50_ in the alamarBlue test was 60 μM, very close to the IC_50_ for cell viability, showing a clear coupling between mitochondrial inhibition and cell death (Figure 3a,c). These experiments were carried out at 24 h incubation; however, rotenone and MPP^+^ inhibited the mitochondrial function earlier after 6 h incubation before inducing cell death. Conversely, 6-OHDA-induced mitochondrial inhibition started at 12 h and it paralleled the time-course of cell death (data not shown).

As an additional test to confirm mitochondrial inhibition, we incubated each toxin for 24 h and checked the cellular ATP content with respect to untreated control (Figure 3b). As expected, we found a dose-dependent drop of ATP amount, with IC_50s_ of 16 nM, 83 μM and 65 μM for rotenone, MPP^+^ and 6-OHDA, respectively. Again, ATP content is commonly used as an indicator of cells viability, but for compounds interfering with cell metabolism alternative techniques are required to correlate ATP reduction to cell death [37]. By comparing the IC_50_ of ATP reduction with the viability test, we can assert that only a small proportion of cells (~5%) were dead at these IC_50_ values in the case of rotenone and MPP^+^, while for 6-OHDA the IC_50_ of the ATP test was similar to the IC_50_ of the vitality test (Figure 3c). 

### 2.4. Effect of Rotenone, MPP^+^ and 6-OHDA on INS-1 β-Cells Morphology 

**Figure 4:**
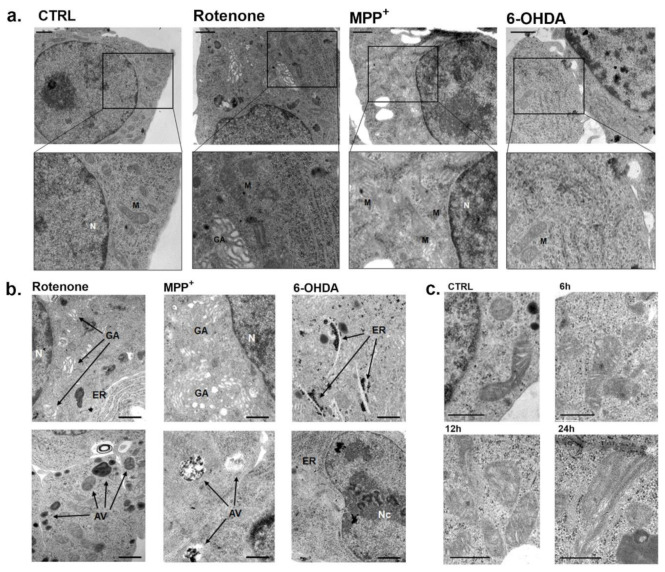
Electron micrographs of INS-1 β-cells exposed to rotenone, MPP^+^ and 6-OHDA. (**a**) Ultrathin sections of INS-1 cells with enlarged zoom in of mitochondria after 6 h incubation with rotenone (15 nM), MPP^+^ (80 μM) and 6-OHDA (65 µM), corresponding to the IC_50s_ of mitochondrial inhibition for each toxin. Scale bars correspond to 1 μm. (**b**) Specific alterations observed after exposure to the IC_50s_ of mitochondrial inhibition for each toxin. Scale bars correspond to 1 μm (**c**) Electron micrographs of progressive INS-1 mitochondrial alterations after exposure with rotenone 15 nM to increasing times (6, 12 and 24 h). All the figures are representative of three different experiments. Scale bars correspond to 500 nm. N: nucleus; Nc: nucleolus; M: mitochondria; GA: Golgi apparatus; ER: endoplasmic reticulum; AV: autophagic vacuole.

The electron microscopic analysis of INS-1 β-cells exposed to these three toxins revealed several morphological alterations. For simplicity, we decided to report our observations at a concentration roughly corresponding to the IC_50_ for mitochondrial inhibition of each toxin and after 6 h incubations, to detect early changes in the mitochondrial structure, when cell death was very low. We could find some isolated cell undergoing necrosis or apoptosis, but the vast majority of cells was alive. Exposure to 15 nM rotenone significantly affected mitochondrial ultrastructure. In fact, many mitochondria were markedly enlarged with dilated disorganized cristae (Figure 4a). Moreover, the endoplasmic reticulum and Golgi apparatus were diffusely enlarged and autophagic vacuoles were common (Figure 4b). The progression of mitochondrial damage was clear with the appearance of paracrystalline inclusions after 24 h exposure with rotenone (Figure 4c). 

Exposure to 80 μM MPP^+^ for 6 h showed similar alterations of mitochondrial ultrastructure together with a remarkable dilation of the Golgi apparatus. In addition, an increased number of autophagic vacuoles was detected (Figure 4a,b). The 6 h incubation with 65 μM 6-OHDA showed a remarkable dilation of the endoplasmic reticulum and large nucleoli in several cells, while mitochondria were not affected (Figure 4a,b).

### 2.5. Effect of Antidiabetic and Antiparkinsonian Drugs and Antioxidants on Rotenone-Induced Toxicity

**Figure 5:**
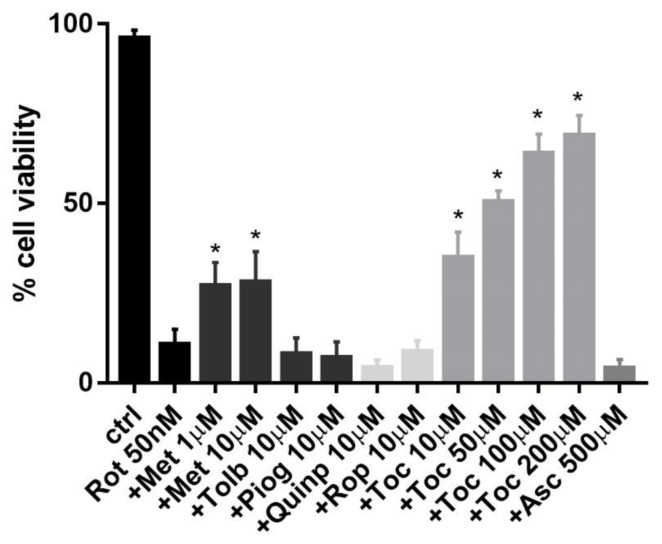
Protective effect of different compounds on rotenone-reduced INS-1 β-cells viability. Cell viability was assessed after 24 h exposure to rotenone 50 nM, with or without the presence of several compounds belonging to different classes of drugs with putative protective characteristics. Data are expressed as mean ± SEM of three independent experiments. * *p* < 0.05 compared to rotenone 50 nM, ANOVA, Dunnett’s *post hoc* test.

After determining the high susceptibility of INS-1 β-cells to rotenone toxicity, we tested several compounds endowed with cytoprotective and antioxidative properties to try to prevent rotenone-induced cell death. Cells were treated with commonly used antidiabetic and antiparkinsonian drugs for up to 10µM concentration, well above their Ki and with the antioxidants α-tocopherol and ascorbic acid up to 500 µM, based on previous works (Figure 5).

Metformin was the only antidiabetic medication to show a partial protective effect either at 1 µM or 10 µM, whereas pioglitazone and tolbutamide showed no change in rotenone toxicity at the same concentrations. The D_2_ dopamine receptor agonists quinpirole and ropinirole showed no protection up to 10 µM. Regarding the use of antioxidants, α-tocopherol protected against rotenone toxicity in a dose-dependent manner starting from 10 µM and it restored cell viability up to 70% at 100 µM, compared to the 10% of cell survival in the presence of the toxin alone, whereas ascorbic acid was non protective up to 500 µM. 

## 3. Discussion

Neurotoxins such as rotenone, MPP^+^ and 6-OHDA are known for their high toxicity on dopaminergic neurons and this has been considered relevant to postulate an association between pesticides and PD. Conversely, the potential vulnerability of pancreatic β-cells for certain environmental toxins has been recently introduced and the epidemiology on this matter is still controversial [6]. This confrontation stems from the intriguing similarity between dopaminergic neurons and β-cells in terms of expression of various elements of the dopaminergic system (e.g., dopamine receptors, enzymes and transporters) and their vulnerability to oxidative stress for the high production of ROS and low expression of endogenous antioxidants [19].

Based on these premises, in the present study, we evaluated the toxicity of these three toxins on the rat-derived INS-1 and mouse-derived MIN-6 β-cell lines. Our results showed a high toxicity of rotenone on β-cells viability and other cellular functions at low nanomolar concentration. For MPP^+^ and 6-OHDA, the IC_50_ for viability were higher in the micromolar range, still showing a vulnerability of β-cells to these toxins.

One possible explanation is that rotenone being lipophilic can easily pass through different cell membranes whereas MPP^+^ and 6-OHDA require DAT that is highly expressed particularly in the dopaminergic neurons [20].

Rotenone is a very potent toxin in the low nanomolar range for several neuronal cell lines, such as SH-SY5Y and PC-12 and for primary mesencephalic dopaminergic neurons [38,39]. In other studies, rotenone was used on β-cells as an inhibitor of mitochondrial activity; however, its toxicity was not analyzed systematically and the IC_50_ for viability was not determined [40,41]. Here, we reported for INS-1 β-cells a high toxicity of rotenone after 24 h with an IC_50_ = 30 nM, which is very similar to that found for dopaminergic-like cell lines. 

**Figure 6:**
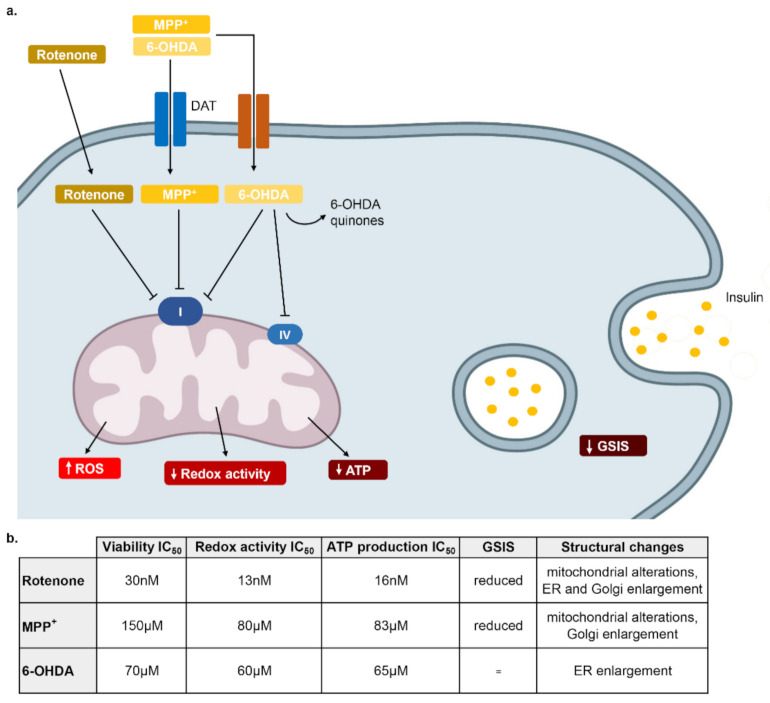
Schematic representation of the mechanisms responsible of rotenone, MPP^+^ and 6-OHDA toxicity in β-cells. (**a**) Rotenone, highly lipophilic, enters the cell freely, it reduces GSIS and it strongly inhibits mitochondrial Complex I, causing an increase of ROS production that is responsible of cell distress. MPP^+^ enters the cell through DAT and other transporters and then it has a mechanism similar to rotenone. 6-OHDA enters the cell through DAT transporter and then it transforms to 6-OHDA quinones responsible of the formation of H_2_O_2_ and other oxidative species. It also reduces mitochondrial complex I and IV activity but it does not affect GSIS. (**b**) Table summarizing the IC_50_ of the three neurotoxins in the different assays and morphological changes analyzed with electronic microscopy.

Intriguingly, in other cell types, such as microglial cells, breast cancer cells and fibroblasts, rotenone was toxic but at higher concentrations, showing a similarity between β-cells and dopaminergic cell lines in terms of high vulnerability to rotenone [42,43,44]. Regarding the mechanism of action, rotenone acts as mitochondrial complex I inhibitor and it has been used as a PD model for both in vitro and in vivo studies [25] (Figure 6). This inhibitory effect is responsible for mitochondrial dysfunction that causes an increase of ROS production and a reduction of cellular bioenergetics. In our experiments, the incubation of INS-1 β-cells with rotenone resulted in mitochondrial functional and structural alterations. In fact, by using the alamarBlue assay as an indicator of the mitochondrial redox activity, we registered a strong reduction of redox activity with an IC_50_ = 13 nM in the presence of the toxin. In addition, along with the reduction of redox activity, the ATP levels also dropped in rotenone-treated cells at similar concentrations. Taken together, these data confirm the inhibitory action of rotenone on β-cells mitochondria, involving complex I, altering cellular bioenergetics and increasing ROS production that is responsible for cell death.

For MPP^+^, we can postulate a similar mechanism as it is a potent inhibitor of the mitochondrial complex I [29], whereas for 6-OHDA the generation of H_2_O_2_ and other oxidative species through its chemical transformation should be responsible for its toxicity together with the inhibition of mitochondrial complex I and IV [21] (Figure 6). The connection between mitochondrial inhibition, determined by redox activity or ATP production and cell death is not simple [45]. In fact, in our experiments the IC_50_ of the two complex I inhibitors, rotenone and MPP^+^, in relation to mitochondrial inhibition was lower (more potent) than the IC_50_ determined in the viability test. Most likely, mitochondrial inhibition requires higher concentrations and longer times to be translated in cell death. In a previous work, it was demonstrated that rotenone toxicity was not related to a moderate ATP depletion but to ROS production, as equivalent ATP reduction induced by 2-deoxyglucose did not cause cell death after 12 and 24 h [46]. These data demonstrate that the amount of ATP reduction is another key factor for inducing cell toxicity. Indeed, as it was shown in the electron micrographs, rotenone and MPP^+^ altered mitochondria ultrastructure, while 6-OHDA did not.

In the presence of rotenone at low nanomolar concentration, many mitochondria were markedly enlarged and damaged with dilated disorganized cristae and the appearance of paracrystalline inclusions was evident. Moreover, the ER and the Golgi apparatus were enlarged and autophagic vacuoles were common. We found comparable evidence for MPP^+^ at high concentration, while 6-OHDA produced alterations mostly to the ER and the nucleus. Similar changes have been previously reported in neuronal cell lines in the presence of these neurotoxins [27,47,48].

Intriguingly, the three neurotoxins caused different effects on GSIS when used at non-cytotoxic concentrations. Whereas MPP^+^ inhibited glucose-stimulated secretory function at 6 and 24 h and rotenone showed the same effect after 24 h, 6-OHDA did not cause any change. Complex I plays a key role in the β-cell secretory pathway, by linking TCA cycle-dependent NADH turnover to the ATP/ADP ratio and membrane depolarization [49]. Our results indicate that inhibition of complex I could be detrimental for secretory function of β-cells. This study was focused on β-cells viability in the presence of these three neurotoxins; however, it would be interesting to investigate if these effects are present on other tissues that are relevant for peripheral insulin resistance.

Based on these data, we decided to investigate the protective activity of several compounds against rotenone, which was highly toxic on β-cells. We decided to test some antidiabetic and antiparkinsonian medications and well-known compounds with antioxidative properties.

In one of the first papers on rotenone by Sherer et al. [46], the toxicity on neuroblastoma cells and dopaminergic neurons was reverted by α-tocopherol, acting as an antioxidant counteracting ROS production. Similarly, in our experiments, α-tocopherol strongly protected INS-1 β-cells against rotenone toxicity in a dose-dependent manner and restored cell viability up to 70% compared to the 10% of cell survival in the presence of the toxin alone. These data confirmed the vulnerability of INS-1 β-cells to ROS due at least in part to low expression of endogenous antioxidants, which can be augmented by using an exogenous source of antioxidants such as α-tocopherol. α-tocopherol is a very potent lipophilic antioxidant and a free radical scavenger, especially at the level of the cell membranes and it protects phospholipids from lipid peroxidation and proteins from oxidation [50,51]. 

Among the antidiabetic drugs tested against rotenone toxicity, we found that metformin slightly reversed this effect. Metformin is the most used antidiabetic compound endowed with pleiotropic activity and it has been found to be an effective cytoprotective agent in many in vitro and in vivo studies. The protective mechanisms of metformin are multifaceted, including antioxidative and anti-inflammatory properties, protection of mitochondria and modulation of autophagy through the AMPK/mTOR pathway [52,53,54]. In addition, it protects from ROS production and increases the expression of antioxidant enzymes, as recently confirmed in rat red blood cells treated with rotenone [55]. Considering the relevance of the oxidative damage in the mechanism of these neurotoxins, future research should analyze oxidative markers induced by ROS production, such as DNA damage, lipid peroxidation and protein oxidation in β-cells, as it has been reported for other cellular systems [46]. In addition, based on the rescue of cell viability in the presence of α-tocopherol and metformin, total antioxidant capacity should be determined by analyzing the relevance of enzymatic and not enzymatic antioxidants.

In conclusion, our data have demonstrated that rotenone is highly potent for reducing INS-1 β-cells viability and altering mitochondrial structure and bioenergetics. MPP^+^ and 6-OHDA show similar effects but at higher concentration. The vulnerability of β-cells and dopaminergic neurons to inhibitors of mitochondrial activity and the protective effect of α-tocopherol and metformin highlight the potential role of mitochondria in the etiopathogenesis of PD and T2D, identifying protective compounds that could be useful for the treatment of both conditions.

## 4. Materials and Methods

### 4.1. Reagents

Rotenone, MPP^+^ iodide, 6-OHDA hydrochloride, metformin hydrochloride, pioglitazone, tolbutamide, quinpirole hydrochloride, ropinirole hydrochloride, α-tocopherol, L-ascorbic acid and trypan blue solution 0.4% and RI-13K Rat Insulin RIA were purchased from Merck Life Science. The Dead Cell Apoptosis Kit with Annexin V-FITC and PI and the alamarBlue Cell Viability Reagent were purchased from ThermoFisher Scientific. For the detection of ATP level, we used the ATPlite 1 step Luminescence Assay System by Perkin Elmer, Inc. (Waltham, MA, USA). 

### 4.2. Cell Culture

INS-1 and MIN6 cells were cultured at 37 °C in humidified atmosphere containing 5% CO_2_. INS-1 complete medium was composed of RPMI 1640 supplemented with 5% heat-inactivated foetal calf serum (FCS), HEPES 1 mM, sodium pyruvate 1 mM, 2-mercaptoethanol 50 μM, streptomycin 0.1 mg/mL and penicillin 100 units/mL. MIN6 cell culture medium was composed of DMEM high glucose, supplemented with 12.5% heat-inactivated FCS, sodium pyruvate 1 mM, 2-mercaptoethanol 50 μM, streptomycin 0.1 mg/mL and penicillin 100 units/mL. The maintenance cultures were passaged twice a week by gentle trypsinization and resuspended in a 75 cm^2^ flask. All the experiments were performed between passage 55 and 70 for INS-1 cells. Cells were seeded at 10^5^ cells/cm^2^, let grow for 48 h and treated for the proper duration of the experiment. 

### 4.3. Cell Viability

Cells were seeded in a 12 well plate and after 48 h were incubated with fresh medium containing different concentrations of the three toxins. The main experiments were performed after 24 h incubation, but a time course on cell viability for each toxin was performed. Upon completion of exposure, cells were trypsinized and mixed with trypan blue. Cells that excluded the dye were counted as viable. Protection experiments were performed with 60 min pre-treatment with the compounds studied followed by 24 h incubation with rotenone. We also confirmed the data with flow cytometry for apoptotic/necrotic cells. After treatment, cells were collected, washed in PBS and resuspended in Annexin binding buffer and stained with FITC-conjugated Annexin V and PI according to the manufacturer’s instructions (Miltenyi Biotec, Bergisch Gladbach, Germany). Flow cytometry of samples was performed using MACSQuant^®^ Analyzer 10 (Miltenyi Biotec). Doublets discrimination was applied before performing DNA content analysis using the MACSQuantify^®^ software (Miltenyi Biotec). The annexin V positive subpopulation was recognized as apoptotic cells, while the PI marked necrotic or late apoptotic cells. The viability experiments were performed at 6, 12, 18 and 24 h, with or without each toxin and non-exposed cells were used as a control. 

### 4.4. Insulin Secretion

Cells were seeded in 24 well plates and after 48 h were incubated with fresh medium containing different toxin concentrations for 6 h and 24 h. Non-exposed cells were used as a control and were incubated for the same time. At the end of the treatment time, cells were washed twice for 10 min with Krebs–Ringer bicarbonate (KRBH) buffer supplemented with 0.5% bovine serum albumin and 2 mM glucose and then incubated for 1 h at 37 °C in 1 mL fresh KRBH buffer containing 2 mM glucose. After that, cells were incubated for 1 h at 37 °C in KRBH buffer containing 11 mM glucose. The buffer was collected for insulin determination and 1 mL of cold acidified ethanol (150:47:3, *v*/*v*, absolute ethanol/H_2_O/concentrated HCl) was added to the cells to extract their insulin content. The plate with acidified ethanol was incubated for 24 h at 4 °C in darkness and the buffer was collected. Insulin was measured by radioimmunoassay according to previous works, using rat insulin as a standard. The sensitivity and the coefficient of variation of the radioimmunoassay were as follows: detection limit 0.13 ng/mL, intra-assay variation 3.3%, inter-assay variation 10.5% [56].

### 4.5. Measurements of Mitochondrial Function

For the measurement of mitochondrial redox alterations, cells were seeded in a 12 well plate and let grow for 48 h. Fresh medium was replaced and treatment was started. Controls only had medium replacement with no treatment. Three hours before the endpoint, 10% alamarBlue Cell Viability Reagent was added to the medium. The 100 μL aliquots were transferred from each well to a 96 well plate for reading. Reading of the assay was performed by a Thermo Scientific Multiskan^®^ Spectrum with a wavelength of 570 nm (and a reference wavelength of 600 nm) as described by the manufacturer. For ATP cell production detection, we seeded cells in a 96-well white plate, let them grow for 48 h and treated them for 24 h with the three toxins. We subsequently added to each well 100 μL of ATPlite 1 step, shook the plate for 2 min at 700 rpm, waited for 10 min to dark adapt the plate and measured the luminescence with BioTek Synergy H1 Hybrid Multi-Mode Reader.

### 4.6. Electron Microscopy

After incubation in fresh medium containing different concentrations of the three toxins, INS-1 cells were fixed in 2.5% glutaraldehyde in 0.1 M phosphate buffer for 20 min at room temperature, washed in 0.1 M phosphate buffer pH 7.3, post-fixed in 0.1% osmium tetroxide in the same buffer pH 7.3 and dehydrated in a graded series of ethanol. In the last phase of dehydration, cells were scraped and the cell suspension was centrifuged to obtain a pellet. Centrifuged pellets were rapidly transferred to propylene oxide and embedded in PolyBed 812 (Polyscience Inc., Warrington, PA, USA). Ultrathin sections were stained with uranyl acetate and lead citrate and observed under a Jeol 100 SX transmission electron microscope.

### 4.7. Statistical Analysis

All results were expressed as mean ± SEM. Statistical significance was evaluated by using analysis of variance (ANOVA) followed by Dunnett’s post hoc test. Data analysis, graphs and dose-response plots were made with GraphPad Prism 7. 

## Data Availability

The data presented in this study are available on request from corresponding authors.
